# DNA nanostructure-based nucleic acid probes: construction and biological applications

**DOI:** 10.1039/d1sc00587a

**Published:** 2021-05-11

**Authors:** Dong-Xia Wang, Jing Wang, Ya-Xin Wang, Yi-Chen Du, Yan Huang, An-Na Tang, Yun-Xi Cui, De-Ming Kong

**Affiliations:** State Key Laboratory of Medicinal Chemical Biology, Nankai University Tianjin 300071 P. R. China yunxilei22@126.com kongdem@nankai.edu.cn; Tianjin Key Laboratory of Biosensing and Molecular Recognition, Research Centre for Analytical Sciences, College of Chemistry, Nankai University Tianjin 300071 P. R. China; College of Life Sciences, Nankai University Tianjin 300071 P. R. China

## Abstract

In recent years, DNA has been widely noted as a kind of material that can be used to construct building blocks for biosensing, *in vivo* imaging, drug development, and disease therapy because of its advantages of good biocompatibility and programmable properties. However, traditional DNA-based sensing processes are mostly achieved by random diffusion of free DNA probes, which were restricted by limited dynamics and relatively low efficiency. Moreover, in the application of biosystems, single-stranded DNA probes face challenges such as being difficult to internalize into cells and being easily decomposed in the cellular microenvironment. To overcome the above limitations, DNA nanostructure-based probes have attracted intense attention. This kind of probe showed a series of advantages compared to the conventional ones, including increased biostability, enhanced cell internalization efficiency, accelerated reaction rate, and amplified signal output, and thus improved *in vitro* and *in vivo* applications. Therefore, reviewing and summarizing the important roles of DNA nanostructures in improving biosensor design is very necessary for the development of DNA nanotechnology and its applications in biology and pharmacology. In this perspective, DNA nanostructure-based probes are reviewed and summarized from several aspects: probe classification according to the dimensions of DNA nanostructures (one, two, and three-dimensional nanostructures), the common connection modes between nucleic acid probes and DNA nanostructures, and the most important advantages of DNA self-assembled nanostructures in the applications of biosensing, imaging analysis, cell assembly, cell capture, and theranostics. Finally, the challenges and prospects for the future development of DNA nanostructure-based nucleic acid probes are also discussed.

## Abstract 2

In recent years, DNA has been widely noted as a kind of material that can be used to construct building blocks for biosensing, *in vivo* imaging, drug development, and disease therapy because of its advantages of good biocompatibility and programmable properties.

## Graphical abstract

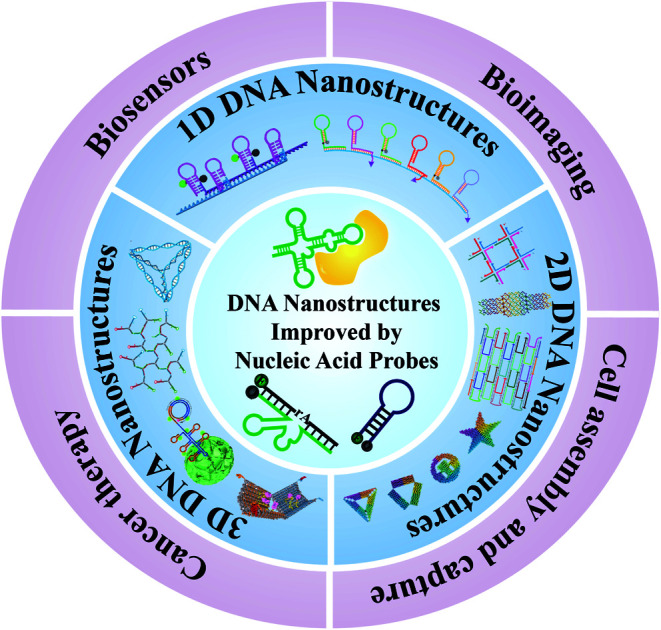

## 1. Introduction

Nucleic acids, including DNA and RNA, are classic biomacromolecules which are well known in relation to genetics and other biological processes.^1^ In recent decades, nucleic acids have also been recognized as materials or probes which could be applied in analytical and biological fields.^2,3^ Due to the advantages of excellent biocompatibility, low cost, ease of synthesis, modification and functionalization, flexible and diverse signal amplification strategies, and modular structures, nucleic acid probes, especially DNA probes, have been widely used for biosensing, bioimaging and medical diagnosis.^4^ However, traditional DNA-based sensing processes are mostly achieved by random diffusion of free DNA probes, which were restricted by limited dynamics and relatively low efficiency. In addition, traditional single-strand DNA probes showed poor cell internalization capability due to their small size, hydrophilic properties, and negatively charged backbone. Even if they enter cells, DNAs are also easily degraded by nucleases before reacting with the target sites. New opportunities are created for the biological applications of DNA probes due to the use of nanomaterials as carriers. The commonly used nanocarriers include organic and inorganic nanoparticles, polymers, hydrogels, liposomes, polymeric micelles, and so on.^5^ They might endow DNA probes with some outstanding features such as enhanced resistance to enzymatic digestion, elevated cell internalization efficiency, and improved probing performance. However, despite the unique properties of these nanomaterials, most of them suffer from obvious cytotoxicity, poor biocompatibility, complicated preparation, and batch-to-batch variations, which seriously limit their practical applications. Different from these exogenous materials, DNA is almost nontoxic and highly biocompatible. Moreover, DNA is endowed with more benefits such as a predictable base-pairing rule, and programmable and controllable self-assembly behaviors. These advantages make DNA promising building blocks to construct pure DNA nanomaterials with predesigned structures. Besides sharing the common advantages of nanomaterials such as high biostability and cell internalization capability, the DNA nanostructures exhibit excellent biocompatibility. More interestingly, they can be easily modified with regular nucleic acid probes or directly designed as sensing probes, thus opening up new paths and opportunities to promote the biological applications of nucleic acid probes.

To date, nucleic acid probes based on DNA nanostructures have shown great potential in biosensing, bioimaging, drug delivery, cell biology and material manufacturing applications. Typical examples of DNA nanostructures mainly include regular shapes such as Y-shaped scaffolds,^6^ DNA tetrahedrons,^7^ polyhedrons,^8^ prisms,^9^ DNA dendrimers,^10^ amorphous structures such as DNA hydrogels,^11^ and complicated programmed DNA nanostructures designed based on DNA origami.^12,13^ All of these structures, with precise designs, can be assembled as confined components with a particular size and shape or expanded into microscopical space. With the development of biochemical technology, DNA modification at a special site became more and more elaborate. Therefore, more and more intelligently designed DNA nanoprobes that exhibit excellent performance in the analysis of small molecules, biomolecules, bio-microenvironments and so on have been developed. Scientists have progressively realized that DNA nanostructures are attractive tools in biomedicine, analytical science, and materials science.

**Fig. 1:**
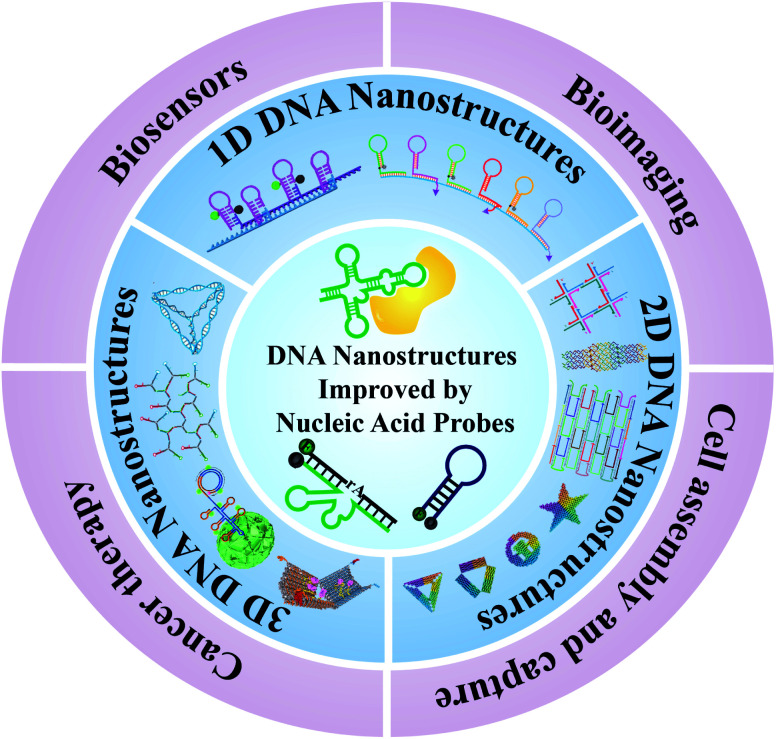
Schematic of DNA nanostructure-based nucleic acid probes: construction and biological applications.

In this review, we will review and summarize the most important advantages in the design of DNA nanostructure-based nucleic acid probes, and their applications in biosensing, bioimaging, biotechnology, and disease diagnosis and therapy (Fig. 1). Firstly, we will introduce the typical DNA nanostructure-based probes according to the dimension classification of nanostructures (one, two, and three-dimensional nanostructures). The major strategies and unique properties of these nanostructures will be clarified and discussed. Secondly, we will introduce the common connection modes between nucleic acid probes and DNA nanostructures. Thirdly, we will review and summarize the most important advances of DNA self-assembled nanostructures in the applications of biosensing, imaging analysis, cell assembly, cell capture, and theranostics. Finally, we will discuss the current challenges and prospects for the future development of DNA nanostructure-based nucleic acid probes.

## 2. DNA nanostructure-based nucleic acid probes

**Fig. 2:**
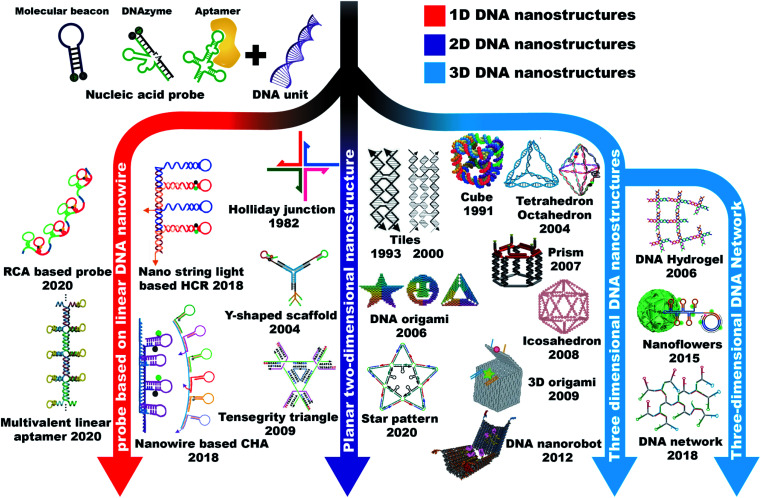
A brief history of the timeline of 1D, 2D, and 3D DNA nanostructures. 1D DNA nanostructures: RCA based probe. Reproduced from ref. 18 with permission from the American Chemical Society, copyright 2020. Multivalent linear aptamer. Reproduced from ref. 22 with permission from Wiley-VCH, copyright 2020. Nano-string light-based HCR. Reproduced from ref. 23 with permission from the American Chemical Society, copyright 2018. Nanowire based CHA. Reproduced from ref. 21 with permission from the Royal Society of Chemistry, copyright 2018. Reproduced from ref. 24 with permission from the American Chemical Society, copyright 2019. 2D DNA nanostructures: Holliday junction. Reproduced from ref. 25 with permission from Elsevier, copyright 1982. Double-crossover (DX) tiles (left) and triple crossover (TX) tiles (right). Reproduced from ref. 26 with permission from the American Chemical Society, copyright 1993. Reproduced from ref. 27 with permission from the American Chemical Society, copyright 2000. Y-shaped scaffold. Reproduced from ref. 28 with permission from the Nature Publishing Group, copyright 2004. Reproduced from ref. 29 with permission from the Royal Society of Chemistry, copyright 2019. DNA origami. Reproduced from ref. 30 with permission from the Nature Publishing Group, copyright 2006. Tensegrity triangle. Reproduced from ref. 31 with permission from the Nature Publishing Group, copyright 2009. Star pattern. Reproduced from ref. 32 with permission from the Nature Publishing Group, copyright 2020. 3D DNA nanostructures: cube. Reproduced from ref. 33 with permission from the Nature Publishing Group, copyright 1991. Tetrahedron. Reproduced from ref. 34 with permission from the Royal Society of Chemistry, copyright 2004. Octahedron. Reproduced from ref. 35 with permission from the Nature Publishing Group, copyright 2004. Reproduced from ref. 36 with permission from the American Chemical Society, copyright 2018. Prism. Reproduced from ref. 37 with permission from the Royal Society of Chemistry, copyright 2007. Icosahedron. Reproduced from ref. 38 with permission from the National Academy of Sciences of the USA, copyright 2008. 3D DNA origami. Reproduced from ref. 39 with permission from the Nature Publishing Group, copyright 2009. DNA nanorobot. Reproduced from ref. 40 with permission from the American Association for the Advancement of Science, copyright 2012. DNA hydrogel. Reproduced from ref. 41 with permission from the Nature Publishing Group, copyright 2006. Nanoflowers. Reproduced from ref. 42 with permission from the Nature Publishing Group, copyright 2015. DNA network. Reproduced from ref. 43 with permission from the Royal Society of Chemistry, copyright 2019.

When talking about a probe, we are considering how the probe can recognize its target. As for DNA probes, one can easily imagine that a piece of DNA strand can recognize the other DNA strand through base pair hybridization to form the classic Watson–Crick double helix structure. So several DNA-based probing strategies were proposed based on the Watson–Crick hybridization, such as fluorescence *in situ* hybridization (FISH),^14^ DNA molecular beacons (MBs),^15^ and toehold-based double-stranded DNA (dsDNA) probes.^16^ Researchers also found that some DNA strands with special sequences can recognize and bind with specific target molecules (termed as DNA aptamers) or catalyze specific reactions (termed as DNAzymes). These kinds of special DNA can be screened *in vitro* through a process called systematic evolution of ligands by exponential enrichment (SELEX).^17^ Ideally, it is possible to obtain DNA aptamers capable of recognizing any kind of target molecule including nucleic acids, proteins, ions, and other small molecules, as well as pH. Moreover, considering that DNA can be easily modified, functionalized and amplified, there is no doubt that DNA probes can be applied widely in multiple fields of chemistry and biology. However, during the course of using DNA probes in cellular analysis, researchers found that barely linear DNA probes cannot efficiently penetrate cell membranes without the assistance of transfection reagents, and they are susceptible to rapid degradation by nucleases in the cellular environment. Hence, it becomes difficult to employ the DNA probes in a living system. Fortunately, besides its functions of genetic regulation and molecule recognition, DNA has also showed excellent properties as a building block of macromolecules. They can assemble into nanostructures with unique shapes and sizes, and scientists were delighted to find that some of these nanostructures can be efficiently internalized by cells without the help of toxic transfection agents. The combination of DNA probes and DNA nanostructures makes the whole thing attractive in cellular applications. Based on the dimensions of DNA nanostructures, this chapter will describe the typical assembling strategies of one-dimensional (1D), two-dimensional (2D), and three-dimensional (3D) nanostructures and discuss the unique properties of different nanostructures (Fig. 2).

### 2.1. 1D DNA nanostructures

1D self-assembled DNA nanostructures generally have a nanowire shape. When traditional free DNA probes are utilized in a reaction system, DNA probes may collide and diffuse randomly in a homogeneous environment. Therefore, when the concentrations of targets, probes or other reagents are extremely low, the analytical performance of the sensing system will be too poor to meet the requirements of researchers. To address this problem, 1D DNA nanowires were introduced. Typically, a DNA nanowire is constructed from two different single-stranded DNA (ssDNA) with functional sites. By confining free nucleic acid probes in the space along the 1D compact DNA nanostructure, the nanowire can hold different probes, thus transforming the regular “intermolecular” reaction into an “intramolecular” mode.^18,19^ It means the probes can maintain a high local concentration, which greatly improves the reaction efficiency and shortens the reaction time.^20^

**Fig. 3:**
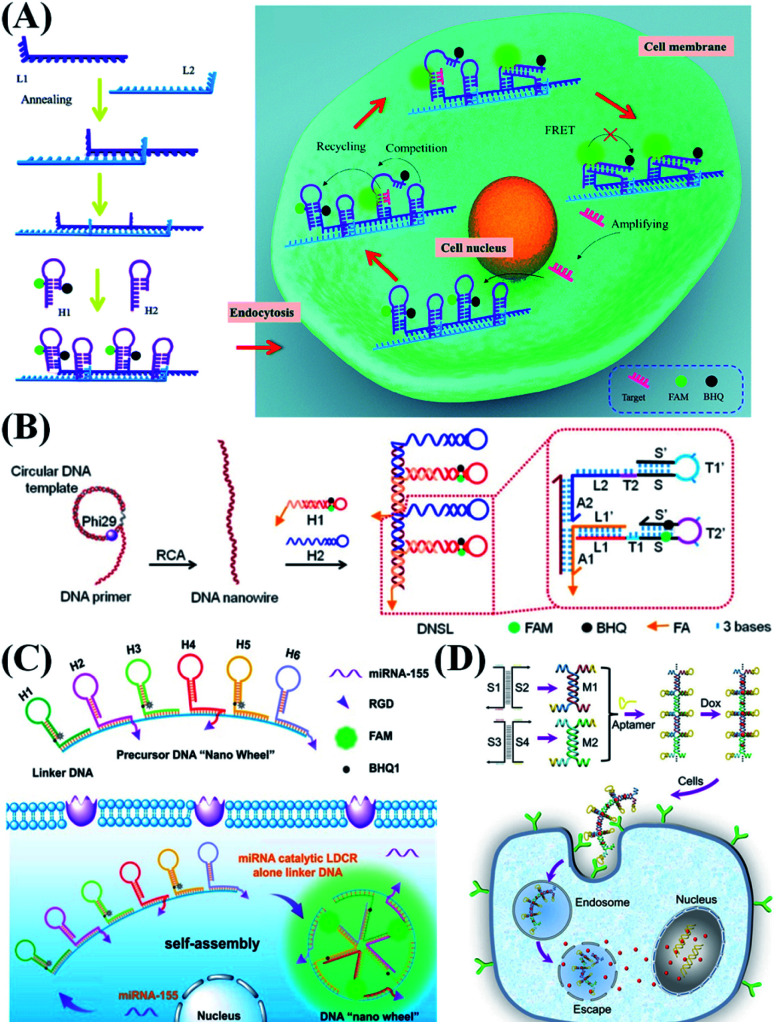
Typical 1D nanostructure-based probe design strategies. (A) DNA nanowire-based localized CHA reaction for *in situ* detection of miR-21. Reproduced from ref. 21 with permission from the Royal Society of Chemistry, copyright 2018. (B) Schematic illustration of a responsive DNA nano-string light generated by RCA. Reproduced from ref. 23 with permission from the American Chemical Society, copyright 2018. (C) Schematic illustration of a miRNA triggered DNA “nano-wheel” for visualizing intracellular miRNA. Reproduced from ref. 24 with permission from the American Chemical Society, copyright 2019. (D) Stepwise assembly of a DNA nanowire for cancer-targeted drug delivery. Reproduced from ref. 22 with permission from Wiley-VCH, copyright 2020.

Wang *et al.*^21^ reported a DNA nanowire-based localized catalytic hairpin assembly (LCHA) reaction for miRNA imaging in living cells (Fig. 3A). In their design, a DNA nanowire concentrates two kinds of hairpin probe in a compact space, which not only increases the local reactant concentrations to shorten the CHA reaction time and improve the sensitivity, but also protects the probes from nuclease degradation and improves their stability during intracellular delivery. Zhang *et al.*^24^ designed a DNA “nano-wheel” to further amplify the fluorescence detection signal during miRNA analysis (Fig. 3C). In the presence of one target miRNA, it can trigger the release of three fluorophores, highly improving the detection efficiency. Through strand replacement recovery, the target miRNA could work as a repeatedly used catalyst to generate more fluorescence. Ju *et al.*^23^ reported a responsive DNA “nano-string light” that realizes rapid and efficient imaging of messenger RNA (mRNA) in living cells *via* accelerating the DNA cascade reaction (Fig. 3B). They used the rolling circle amplification (RCA) reaction to produce long single strands as the template, and two DNA probes could successfully hybridize on the template to form a nanowire-like structure. The reaction time is 6.7-fold shorter than that of the conventional DNA cascade reaction. Modification of cell-specific aptamers on DNA nanowires has also been performed (Fig. 3D). The inner stem is composed of multiple connected DNA double helices, and the outer branch is composed of regularly arranged upright hairpin aptamers. In recent years, Lu *et al.* used this kind of nanowire as a targeted therapeutic agent.^22^ It demonstrates an ideal therapeutic vehicle for tumor-targeted imaging and inducing tumor cell apoptosis.

### 2.2. 2D DNA nanostructures

Compared with 1D DNA nanostructures, 2D nanostructures expand the effective territory for molecular fixation. Meanwhile, the signal molecules can be modified at the specified spots to make their movement controllable, thus enabling better study of intermolecular interactions.

#### 2.2.1. Y-shaped scaffold

**Fig. 4:**
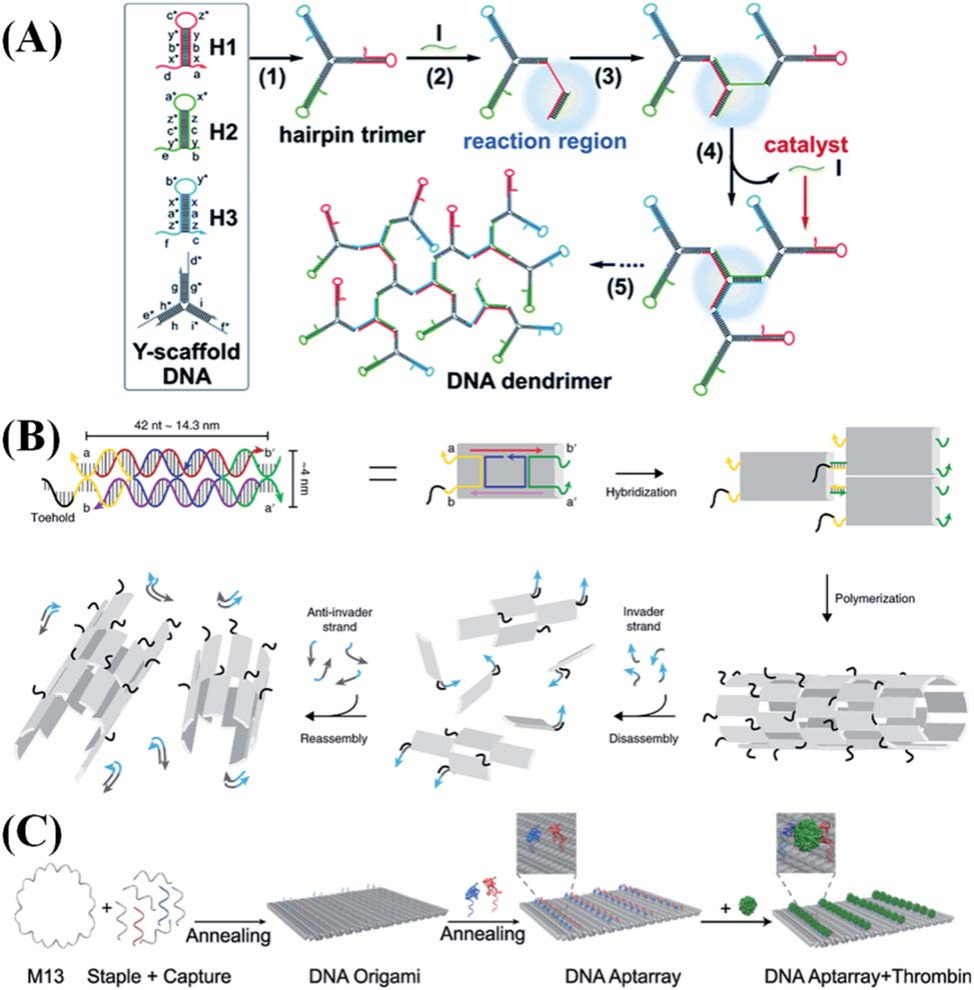
Typical 2D nanostructure-based probe design strategies. (A) Illustration of the tripartite Y-shaped DNA probe and bCHA circuit for RNA imaging. Reproduced from ref. 43 with permission from the Royal Society of Chemistry, copyright 2019. (B) Schematics of DNA nanotube self-assembly and proposed control mechanisms. Reproduced from ref. 52 with permission from the Nature Publishing Group, copyright 2019. (C) Schematic illustration of the construction of thrombin aptamer-loaded nanoarrays by DNA origami. Reproduced from ref. 60 with permission from the Nature Publishing Group, copyright 2021.

A Y-shaped scaffold is a tripartite DNA probe that is composed of three DNA strands.^28^ Such a tripartite DNA probe can connect three different functional nucleic acid probes to form an integrated nanostructure. A Y-shaped scaffold is one of the smallest DNA structures with tripartite functions, which endows it with many advantages like easy operation, low cost, and improved performance in live organelles. Besides, the Y-shaped scaffold can ensure that all three different sensing probes are delivered into the cells simultaneously in equal amounts, thereby reducing the signal interference and improving the accuracy of multiple-biomarker detection.^6^ Bi *et al.*^43^ reported DNA dendrimers which were constructed by dynamic self-assembly of a Y-shaped scaffold based on branched catalytic hairpin assembly (bCHA) (Fig. 4A). Compared with traditional CHA, bCHA introduces a proximity “diffusion effect”, which significantly improves the signal amplification efficiency. Jiang *et al.*^29^ linked folate receptors to a Y-shaped scaffold to enhance the cancer cell specificity and then used the hybridization chain reaction (HCR) for ultra-sensitive RNA detection in cells. They also developed a tripartite DNA probe that enables CHA for fluorescence imaging of RNA in living mice.^44^ By connecting three different miRNA recognition sequences to a Y-shaped scaffold, it can realize the *in situ* multiple detection of serum exosomal miRNAs for breast cancer diagnosis. Moreover, the Y-shaped scaffold linking functional probes can be applied in tumor diagnosis and treatment.^45^ Wang *et al.*^46^ developed a multivalent connection method for extracellular pH regulation. Under acidic extracellular conditions, aptamer-based Y-shaped scaffold structures, which were originally mono-disperse, might tend to cross-link with each other by forming intermolecular i-motif structures. Valence DNA assembly can be applied not only for highly sensitive diagnosis, but also for efficient drug delivery, and achieving the targeted inhibition of tumor cells.^47^

#### 2.2.2. DNA tiles

In 1982, Dr Nadrian Seeman proposed a cross-shaped Holliday structure based on Watson–Crick base pairing (Fig. 2).^25^ Different from the conventional linear dsDNA, it has more extensible directions and a more rigid backbone, making it a promising block unit to synthesize more complex structures. This artificial four-arm connection extends DNA as a nanomaterial beyond one dimension. In 1993, Seeman's group made improvements based on the four-arm junction, arranging two double helix structures side by side as one arm, and introducing a crossover inside it, thus forming a more stable tile structure called double crossover (DX) tiles.^26^ The regular Holliday junction is characterized by a single crossover between two DNA duplex arms, while a DX tile has two inter-arm crossovers. Therefore, DX tiles are not only more rigid than Holliday junctions but also twice as rigid as linear dsDNA. DX tiles with sticky ends have the amazing properties of controllable size and high nano-mechanical strength. Further research has promoted the tile structures to triple crossover (TX) tiles,^27^ paranemic crossover (PX) tiles,^48^ multi-point stars^38,49^ and other patterned periodic DNA lattice tiles.^50,51^

As early as the 1990s, DX tile-based patterns were used for the synthesis of DNA nanotubes. These artificial DNA nanotubes can be customized and modified to achieve biomimetic functions including ion or molecular channels, bioreactors, drug delivery, and biomolecular sensing. Elisa Franco's group^52^ reported dynamically controlled self-assembled DNA nanotubes. All DNA tiles include toeholds that can be controlled by other input DNA or RNA strands (Fig. 4B), which allow the disassembly and reorganization of nanotubes to be controlled through invader and complementary anti-invader molecules. In addition, a variety of input signals (such as ultraviolet light,^53^ pH,^54^ transcription signal,^55^*etc.*) can activate the disassembly or recombination of the DNA-based nanotubes. These studies contribute to the development of responsive nucleic acid materials. Other tiles containing nucleic acid probes or aptamers have also been reported for biosensing and disease treatment.^56^

#### 2.2.3. DNA origami

Before 2000, DNA nanostructures were usually assembled by annealing DNA strands at a strict stoichiometric ratio. The preparation process is laborious and inefficient. Meanwhile, the constructed DNA nanostructures always have limited size and inerratic shape. In 2006, the invention of DNA origami technology solved most of the limitations in the design and construction of 2D DNA nanostructures.^30^ The basic principle behind DNA origami is the same as that described in Seaman's DNA tiles, that is, to create fixed junctions or crosses between DNA helices and bind them together to form a structure. In a typical DNA origami, many short ‘staple’ chains are used to assist the folding of a long ‘scaffold’ DNA strand to create arbitrarily complex shapes. Specifically, a 7249-nt M13 genomic ssDNA is folded and filled into the required frame shape, and then hundreds of unpurified synthetic short chains are used as anchoring scaffolds. Origami shapes typically have a diameter of ∼100 nm, or a molecular weight of ∼5000 kDa, which is about 20-fold higher than that of a typical TX tile. The DNA origami technology achieves a high yield of the designed structure, avoiding the multiple requirements for sequence optimization, purification, and strict stoichiometry of the constituent chains. Furthermore, non-periodic 2D structures of arbitrary complexity, such as rectangles, smiley faces, stars, and other design patterns, were produced.^30^ In most of the DNA origami protocols, the DNA nanostructures that were constructed relied on Mg^2+^ concentrations as high as at the mM level to compensate for the electrostatic repulsion between neighboring DNA helices.^57,58^ However, the Mg^2+^ concentrations in related biological media (such as urine or serum) are not high enough. Although some physiologically abundant monovalent cations such as Na^+^ or K^+^ were also reported to stabilize DNA origami structures, they are less efficient at holding the whole structure. Linko's group studied the stability of DNA origami structures in low-concentration Mg^2+^ buffer.^59^ By rationally selecting the components of the buffer and considering superstructure-dependent effects, the structural integrity of a given DNA origami nanostructure can be maintained in conventional buffers even supplemented with very low Mg^2+^ concentrations in the μM range.

With the particular design, nucleic acid probes can be placed at a precise distance in the DNA origami structure. Thus, by utilizing the resultant specific geometric structures of origami, biophysical characterization of the interaction of biomolecules can be performed. Fan *et al.*^61^ labeled a nucleic acid probe at a designated position of the DNA origami structure to make it movement-controllable. This integrated structure was then applied to simulate the spatial control distribution of PAM (protospacer adjacent motif) in chromatin, revealing the best catalyzing performance of CRISPR/Cas9, a novel genomic tool.^62^ Origami can also mark molecular beacons or aptamers for biosensing and tumor treatment.^63,64^ Ding *et al.*^60^ used DNA origami as a template to assemble thrombin-bound aptamers with precise nano-spatial control (Fig. 4C). By optimizing the type, distance and number of aptamers, they proved that DNA origami-based aptamer assemblies can specifically and efficiently bind thrombin molecules, thereby inhibiting their coagulation function.

### 2.3. 3D DNA nanostructures

3D DNA nanostructures play a vital role in DNA nanotechnology. They have the most versatile shapes because of the 3D precise orientation at the nanoscale. Despite the relatively difficult manufacturing methods, they show the most advanced design concepts, which allows them to be widely used in biosensing and nanomaterial science.

#### 2.3.1. DNA tetrahedron nanostructures

**Fig. 5:**
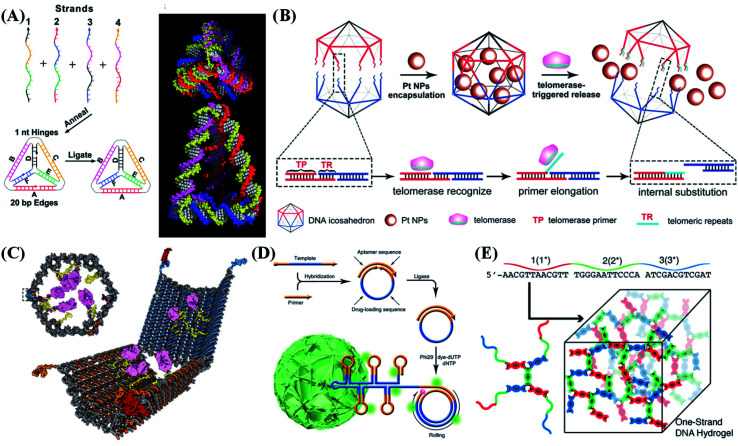
Typical 3D nanostructure-based probe design strategies. (A) Design of a DNA tetrahedron formed by annealing four oligonucleotides. Reproduced from ref. 69 with permission from the American Association for the Advancement of Science, copyright 2005. (B) Construction of a telomerase-responsive DNA icosahedron for platinum delivery. Reproduced from ref. 70 with permission from Wiley-VCH, copyright 2018. (C) Design of an aptamer-gated DNA nanorobot. Reproduced from ref. 40 with permission from the American Association for the Advancement of Science, copyright 2012. (D) Design of self-assembled multifunctional DNA nanoflowers. Reproduced from ref. 42 with permission from the Nature Publishing Group, copyright 2015. (E) Base pairing of multidomain DNA strands to assemble a one-strand DNA hydrogel. Reproduced from ref. 71 with permission from Wiley-VCH, copyright 2016.

A DNA tetrahedron is one of the classic 3D nanostructures most widely used in biosensor design and drug delivery. As long as it has a precise design, a DNA tetrahedron can be completely assembled from short oligonucleotides *via* a simple one-pot annealing process. In 2004, Turberfield *et al.*^34^ first proposed the single-step synthesis of a DNA tetrahedron. Briefly, four equimolar ssDNAs are mixed in a buffer, heated at 95 °C for 5 minutes, and then quickly chilled to room temperature to complete the DNA tetrahedron synthesis (Fig. 5A). In a typical DNA tetrahedron, each ssDNA contains three blocks, which can hybridize with three other strands to complete the assembly of a triangle. Compared with 1D linear or molecular beacon probes, DNA tetrahedrons have stronger mechanical strength. Meanwhile, DNA tetrahedrons have the characteristics of high programmability, low cost, good stability, and high structural strength, which ensure their application prospects in the fields of biosensors, targeted delivery carriers, and molecular biological detection.

DNA tetrahedral nanostructures can construct various scaffold logic gates (INH, XOR, AND and OR). A series of programmed DNA tetrahedron nanostructures containing dynamic sequences that are responsive to protons, metal ions (such as Hg^2+^), small molecules (such as adenosine triphosphate, ATP), and complementary nucleic acid strands (such as miRNA and T7 RNA transcription) have been designed.^66–68^ These DNA nanostructure-mediated logical computations can simultaneously detect biomarkers of disease and control the *in vivo* release of small molecules. DNA tetrahedron-linked hairpin probes can be used to construct a multifunctional optical sensing platform for multiple analyses of miRNAs, endonucleases, and small molecules.^72^ The DNA tetrahedral framework can also promote folding dynamics and improve the thermodynamic stability of aptamers. The binding affinity of the aptamer is increased by ∼3 times after being modified on a DNA framework.^73^ In addition, DNA tetrahedra with tetravalent states can also be designed and covalently bonded to form various high-order nanostructures.^74,75^

#### 2.3.2. DNA polyhedron nanostructures

In addition to the tetrahedrons, 3D DNA polyhedron nanostructures, such as cubes,^33,76,77^ octahedrons,^35,36^ dodecahedrons, and icosahedrons, have also been reported.^8,38^ These polyhedrons have the following advantages over tetrahedrons: (i) more recognition chains can be connected in a compact nanostructure. These recognition strands are close to each other with a large local concentration, thus greatly increasing the reaction rate. (ii) The higher the number of polygon edges, the more flexible the design of each edge, thus making it more programmable. (iii) The polygonal cavity has a greater utilization rate. It can be used to encapsulate other kinds of molecule and even nanoparticle, which has broad application in bioimaging and drug delivery therapy.^70^

Zhang *et al.*^76^ reported hairpin-based DNA cubes for efficient and reliable fluorescence resonance energy transfer (FRET) imaging of miRNA in living cells. Due to the spatial-confinement effect of the DNA nanocube, the miRNA-initiated localized hairpin DNA cascade amplification reaction was significantly accelerated (7-fold faster), and the detection efficiency also greatly improved (2.6-fold higher). In addition to speeding up the reaction rate, DNA cubes can also be used for molecular screening. Tan *et al.*^77^ reported a DNA cube with a cavity-tunable nucleic acid chamber for size-selective molecular recognition. The proposed system consists of functional nucleic acid probes (such as DNAzymes, aptamers, and molecular beacons) which are wrapped in the inner cavity of the DNA cube. Therefore, small target molecules can enter the cube through effective molecular recognition, while large molecules are prohibited. Due to the protection provided by the DNA cube, nucleic acid probes are effectively protected from nuclease degradation and nonspecific protein binding, thereby providing a high degree of stability and accuracy for intracellular sensing. Yang *et al.*^35^ reported a DNA octahedron-based fluorescent nanoprobe that can be applied for detecting and imaging two tumor-related mRNAs in living cells. The DNA icosahedron designed by Gu's group can respond to telomerase in tumor cells and accurately release nanomedicines loaded in the cage, thereby enhancing the anti-cancer effect of nanomedicines towards drug-resistant cancer (Fig. 5B).^70^ This precisely designed DNA icosahedron can be extended to various targeted anticancer drugs. Based on this, a 3D DNA molecule transfer strategy was developed.^8^ A controllable DNA chain pattern can be transferred from the DNA icosahedral cage to the surface of the wrapped gold nanoparticle (AuNP). Through this direct transfer strategy, the amount and position of DNA on the surface of AuNPs can be regulated simultaneously, further expanding the applications of DNA nanotechnology to nanolithography.

DNA prisms, similar to tetrahedrons and polyhedrons, have the advantages of other DNA nanostructures.^37^ DNA prisms are usually constructed as triangular prisms, pentagonal prisms and hexagonal prisms. Li *et al.*^78^ reported a lysosome-recognizing DNA prism nanodevice that can sense the environmental components around lysosomes (protons and ATP) and complete the calculation of cellular DNA. The designed DNA prism logic device can be applied to controllable drug release and disease treatment. DNA prisms are also used in the sensing of some neurotransmitters.^79^ A DNA-nanoprism with aptamer-based “turn-on” fluorescent sensory modules provided a robust and sensitive tool for cell-surface-targeted imaging of neuromodulations.

#### 2.3.3. 3D DNA origami

In 2009, the geometries of DNA origami-based structures were extended from 2D to 3D.^12,80^ Anderson and colleagues created the famous DNA box (42 nm × 36 nm × 36 nm) assembled from six 2D origami sheets (Fig. 2).^39^ In their strategy, the scaffold strand was divided into several parts to form an individual planar substructure. By connecting adjacent planar sub-structures, a series of 3D DNA origami structures were formed. In addition, inserting or deleting base pairs at particular positions could create twisted or curved 3D shapes.^81^ In order to facilitate the design of 3D origami technology, a computer-aided design platform, caDNAno, was developed.^82^ This platform enables simple and rational design for more complicated DNA origami structures. However, an obvious barrier to the application of caDNAno arises from its limitations on the recognition of the crystal lattice, which makes it practically impossible to design material-effective, open, hollow or porous nano-scale conformations. Therefore, advanced wireframe-like DNA structures have attracted great attention.^83^ Another programming platform called vHelix provides an intuitive top-down design and thus models the platform for wireframe DNA replication of user-defined 2D and 3D objects.^84^ Using an applicable meshing tool to transform the designed model into a triangular, polyhedral wireframe mesh, the scaffold strand was folded into the wireframe DNA nanostructure in the design after further algorithms and optimizations. The remarkable development of the wireframe DNA structure design method provides more complex graphics and expands the prevalent shape space.

Most recently, DNA origami technology has been moving towards macroscopic structures.^13,80^ The original sizes of the reported DNA origami are mainly limited from 5–10 nanometers to ∼200 nanometers. Assembling DNA structures into larger sizes (micrometers to millimeters) remains a huge challenge. So far the general method is to expand and elongate the scaffolds or staples which will be used as the initial material to create DNA origami structures. Larger scaffolds can be obtained by the DNA polymerization reaction or extracting larger genomes from bacteriophages. Recently, Wagenbauer *et al.*^85^ reported the largest discrete DNA origami-based structure in solution. Compared to the canonical DNA origami structures which usually have a molecular weight of a few MDa, this new structure has a 1000-fold increase in size thus reaching the GDa-scale. On a macroscopic surface area, Xin *et al.*^86^ recently obtained the biggest DNA origami lattice. They reported that they have fabricated fairly homogeneous DNA origami lattices with an area of ∼20 cm^2^, consisting of approximately one trillion origami structures. Fan and Yan *et al.*^87^ developed the “meta-DNA” (M-DNA) strategy and realized the static and dynamic assembly of various submicrometre-to-micrometre-sized DNA structures. A six-helix DNA origami bundle was used as the basic building block of M-DNA to construct meta-multi-arm junctions, 3D polyhedrons and various 2D/3D lattices, among others. With this expanded-shape device, one can create almost arbitrary structures that may be interesting. Some of the striking examples can be used as sensors, for computing, or as drug capsules.^88,89^

Micro-robots have always been an important field of robotics research. DNA nanorobots have attracted the intense attention of researchers.^90^ Church *et al.*^40^ reported an autonomous DNA nanorobot that can transport molecular payloads to cells, sense the conditionality of the cell surfaces, trigger activation inputs, and reconfigure its structure for payload delivery (Fig. 5C). Unlike mechanical robots in the traditional sense, the key technology applied for DNA nanorobot construction is DNA origami. Based on DNA origami, some controllable mechanisms are integrated, so that the DNA materials work as not only the backbone structures of the nanorobots, but also the fuel to promote them.^91^

#### 2.3.4. DNA nanoflowers

The synthesis of traditional nanostructures based on the Watson–Crick base-pairing principle has many limitations, such as the complex synthesis process, strict molar ratio, and large material concentration requirements. Many strategies have been proposed to overcome these obstacles. Tan *et al.*^42^ reported a multifunctional DNA nanoflower structure. In contrast to DNA nanostructures that rely on Watson–Crick base pairing, DNA nanoflowers are self-assembled by using long ssDNAs as building blocks, which are prepared by the RCA reaction (Fig. 5D). The assembly of DNA nanoflowers is driven by liquid crystallization and dense packaging of building blocks. By artificially changing the RCA template sequence, different functional nucleic acids, including antisense drugs, aptamers, biological imaging agents and drug-loading sites, can be easily packaged into DNA nanoflowers.^92^ Simultaneously, by changing the time of the RCA reaction, it is possible to prepare nanoflowers with reconfigurable sizes from ∼200 nm to ∼2 μm. Because DNA nanoflowers consist of tightly packed long single strands, they exhibit extremely high resistance to nuclease degradation, denaturation, or dissociation at even very low concentrations. By integrating cancer cell-targeting groups, aptamers, fluorophores, and drug-loading sequences into DNA nanoflowers,^93^ the obtained multifunctional DNA nanoflowers can be further used for bioimaging, selective cancer cell identification and targeted anticancer drug delivery. At the same time, they are expected to play promising roles in a variety of biomedical applications.^94^

#### 2.3.5. DNA hydrogel

As a special member of DNA nanostructures, DNA hydrogel is a new type of unconstrained structure with a 3D network. Because of its low toxicity and programmability, it has attracted intense attention. Conventional DNA hydrogels usually use polyacrylamide, poly(propylene oxide), or poly(*N*-isopropyl acrylamide) as the backbone, and functional DNAs (such as aptamers or DNAzymes) as crosslinkers.^95^ To avoid the introduction of toxic reagents and improve the biocompatibility of hydrogels, pure DNA hydrogels have also been prepared *via* complementary base pairing using DNA nanostructures (*e.g.*, T-, X-, and Y-shaped scaffolds) as building blocks.^41^ By changing the initial concentration of the DNA cross-linking agent, the size of DNA hydrogel can be finely tuned in the nanometer range, which is beneficial for intracellular transmission and imaging applications (Fig. 5E). Hydrogel nanocomposites can also respond to external stimuli (such as temperature, DNA strands, pH, or light) to generate signals for sensitive detection, while triggering the dissociative release of the loaded cargo.^96^

English *et al.*^97^ utilized the programmability of a CRISPR/Cas12a nucleic acid probe to activate DNA hydrogels. When the Cas12a/crRNA binary complex was added, the CRISPR system was activated to cleave ssDNA in the hydrogel, thereby transforming biological information into changes in material properties. Such programmable CRISPR-responsive smart nanomaterials are expected to be used in tissue engineering and molecular diagnosis. Jiang *et al.*^98^ constructed a DNA hydrogel by using protein scaffolded DNA tetrads, which are prepared by binding four biotinylated hairpin DNA probes with a streptavidin protein, as building blocks. Target miRNA triggers the crosslinking hybridization chain reaction (cHCR) of the building blocks, generating cross-linked hydrogel network clusters and thus providing high sensitivity and spatial resolution for target miRNA imaging. Such protein scaffold DNA tetrads may provide an effective strategy for nucleic acid delivery, sensitive biomarker imaging, and related theranostics. This kind of DNA hydrogel structure has also been applied in the detection of telomerase^99^ and aptamer-targeted tumor imaging.^100^

## 3. Connection modes between nucleic acid probes and DNA nanostructures

1D, 2D, and 3D DNA nanostructures can all anchor nucleic acid probes for biosensing, bioimaging and disease treatment. This chapter will mainly discuss the main methods researchers employed to couple the nanostructures with nucleic acid probes. According to the number of nucleic acid probes attached to the nanostructure, DNA nanostructure-based nucleic acid probes can be divided into two categories: monovalent and multivalent. In monovalent probes, one nanostructure binds with only one nucleic acid probe. This monovalent binding has been commonly used in the fields of biosensing and bioimaging due to the simple construction operation. However, monovalent probes may cause poor recognition ability, a slow reaction rate and a weak signal output. To solve these problems, DNA nanostructure-based multivalent nucleic acid probes have been developed by simultaneously linking multiple nucleic acid probes to a DNA nanostructure. Multivalent nucleic acid probes can improve the sensitivity of the corresponding biosensing systems, and strongly capture targets *via* the synergy of multiple binding sites.^101^ This chapter focuses on the preparation methods of multivalent probes, including Watson–Crick complementary base pairing and click chemistry conjugation reactions.

### 3.1. Watson–Crick base pairing

**Fig. 6:**
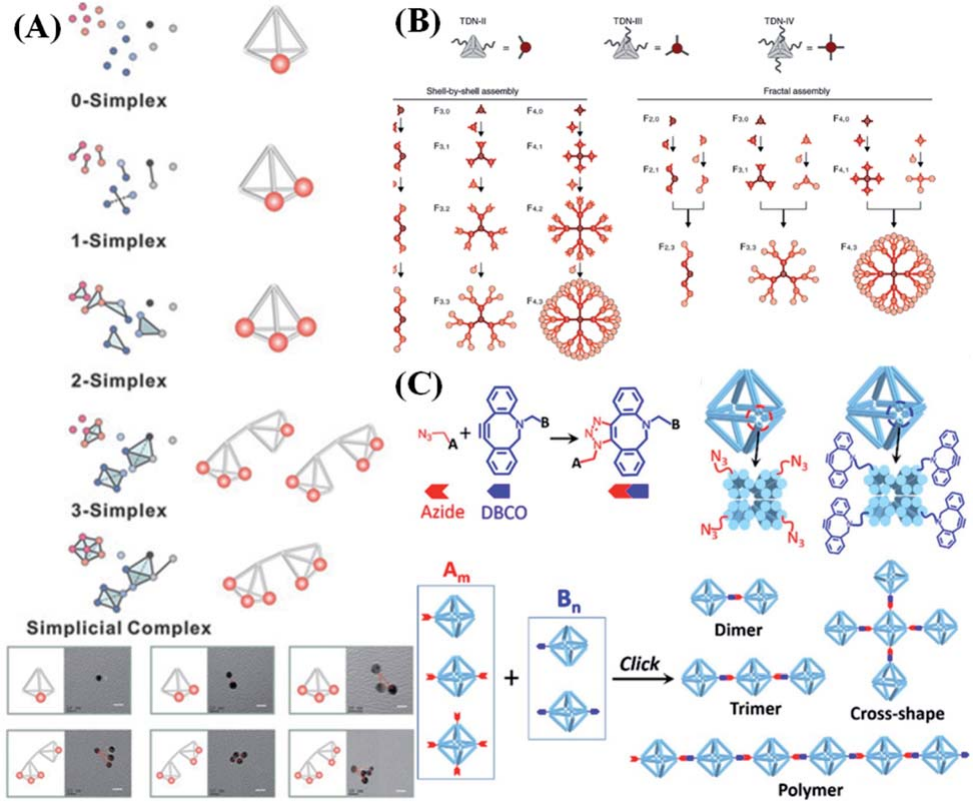
Connection modes between nucleic acid probes and DNA nanostructures. (A) Multivalent DNA framework-based topological cell sorters. Reproduced from ref. 103 with permission from Wiley-VCH, copyright 2020. (B) Fractal DNA frameworks with precise node numbers and molecular weights encoding quantized fluorescence states. Reproduced from ref. 104 with permission from the Nature Publishing Group, copyright 2020. (C) Click reaction with the designated valences for generating controlled architectures. Reproduced from ref. 106 with permission from the American Chemical Society, copyright 2019.

Through precise regulation of the valence state, the biosensing performance (detection sensitivity and dynamic range) of nanoprobes can be well regulated.^74^ The most common strategy to anchor nucleic acid probes on DNA nanostructures is to produce a dsDNA linker between them *via* Watson–Crick base pairing. In this way, Fan *et al.*^75^ reported a valence-controlled framework nucleic acid signal amplifier to recruit special targets such as nucleic acids, proteins and inorganic nanoparticles. Such valence-controlled signal amplifiers can also greatly enhance the quantification ability of electrochemical biosensors, enabling ultra-sensitive detection of tumor-related circulating free DNA (cfDNA). By simply alternating the binding site number on a DNA nanocube, different numbers and different types of nucleic acid probe can be precisely linked based on the high predictability of Watson–Crick base pairing. The as-prepared valency-controlled spherical nucleic acid probes have tunable biosensing properties, such as response kinetics, detection sensitivity and response range.^102^*Via* DNA hybridization, Zhu *et al.*^103^ linked different numbers of ligands (*e.g.*, aptamers) to the vertices of a DNA tetrahedron and DNA tetrahedron dimer, thus achieving the topological control of stoichiometry and spatial arrangement of ligands (Fig. 6A). The as-prepared multi-ligand topological device changes the nature of molecular recognition by inducing receptor aggregation, resulting in a significant (∼10-fold) increase in binding strength between ligands and tetrahedrons. Moreover, the precise engineering of topological complexes formed by tetrahedrons can easily be transformed into an effective binding control device for cell patterning and a cell-binding strength control device for cell sorting. In addition, the use of multivalent DNA frameworks can also topologically encode fluorescence states for multiplex detection of low-abundance targets (Fig. 6B), thus achieving high-throughput detection.^104^ Interestingly, adjusting the ratio of DNA nanostructures and aptamers through regulating the valence state can help to enhance cellular uptake of nanoparticles.^105^

### 3.2. Click chemistry

In addition to complementary base pairing, researchers have also successfully applied chemical reactions to manipulate the assembly of DNA nanostructures. The use of covalent bonds formed by chemical reactions to permanently connect DNA objects provides a simple, efficient and stable molecular connection method. Gang *et al.*^106^ reported a method of using copper-free click chemistry between azide and dibenzocyclooctyl (DBCO) moieties to assemble nanostructures with directionally defined valences (Fig. 6C). Similar to complementary base pairing, the azide and alkyne portions are specifically anchored on the designated vertices of the DNA framework to provide chemically reactive nanostructures with different valence states. By introducing click chemistry molecular reagents, DNA building blocks with a well-defined valence and reactivity provide a new platform for designing and generating structure-defined and covalently bonded nanostructures.

## 4. The applications of DNA nanostructure-based nucleic acid probes

DNA nanomaterials have been widely used in many fields because of their excellent programmability, biocompatibility and stability. The applications of DNA nanostructure-based nucleic acid probes can be divided into several categories, including (i) biosensing, which uses the signal conversion and amplification capabilities of DNA nanostructures to detect multiple types of biomarker *via* electrochemical or fluorescence signal outputs, (ii) bioimaging, which uses DNA nanomaterials as the carriers of nucleic acid probes to trace the location and concentration of intracellular biomolecules such as ATP, cytochrome C, DNA, miRNA, *etc.*, (iii) cell assembly and cell capture, which use functionalized DNA nanostructures to capture cancer cells, followed by cell screening or cell operation to help to reveal cell pathways and provide new directions for medical therapy, and (iv) theranostics, which uses DNA nanostructures for highly efficient disease diagnosis and targeted drug delivery. In summary, the introduction of DNA nanostructures has greatly promoted the applications of nucleic acid probes in analytical chemistry, chemical biology, medical chemistry and so on.

### 4.1. Biosensors

Biosensors can convert biological events into recordable signals. DNA nanostructures can bind with nucleic acid probes, constructing biosensors that can detect various targets such as nucleic acids, metal ions, small molecules, proteins, and cells, as well as pH. To date, DNA nanostructure-based nucleic acid probes have been widely used in biosensing applications. In the presence of targets, the biosensor will accurately capture the target and convert the molecular recognition event to a detectable signal. One challenge in biosensing research is how to promote the maximum interaction between targets and probes as well as how to prevent unnecessary probe agglomeration caused by the irregular distribution of probes on the sensing surface. Properly designed DNA nanostructures have proven to be particularly appropriate to solve this problem. The distance between the probes can be reasonably programmed by using the DNA nanostructure. In recent decades, various types of DNA nanostructure-based biosensor have emerged and been broadly applied. This part will focus on those biosensors based on electrochemical and fluorescent transducers, discussing how researchers employ them for the analysis of different biomolecules.

#### 4.1.1. Electrochemical sensing

Conventional DNA-based electrochemical sensors usually anchor ssDNA probes on the surface of electrodes. Specific target recognition will cause the allosteric conformational change of DNA probes, thus triggering the change of electrochemical signals. The flexibility of ssDNA makes it difficult to control the density and orientation of the probes on an electrode surface, which might seriously affect the reaction efficiency between capture probes and targets. To overcome this drawback, Fan *et al.*^65^ reported a strategy using tetrahedral DNA probes as an alternative to ssDNA probes. Compared with conventional ssDNA probes, tetrahedral DNA probes have advantages in the following aspects: first, three –SH modified vertices of the DNA tetrahedron can easily anchor on the electrode surface, greatly improving the binding stability of probes on the electrode surface; second, the whole structure has a rigid backbone, which ensures that the nucleic acid probe modified on the fourth vertex of the DNA tetrahedron can be well orientated to avoid entanglement between two probes; third, the modification of the DNA tetrahedron on the surface has a good passivation effect on the electrode, which can prevent non-specific adsorption of small molecules. Considering that the lateral spacing between the probes is also one of the important factors affecting the efficiency of the sensor, DNA tetrahedrons with stem length ranging from 2.4 to 12.6 nm were designed to modulate the probe's distance.^65^ This research provides the possibility of developing more sensitive electrochemical sensors.

**Fig. 7:**
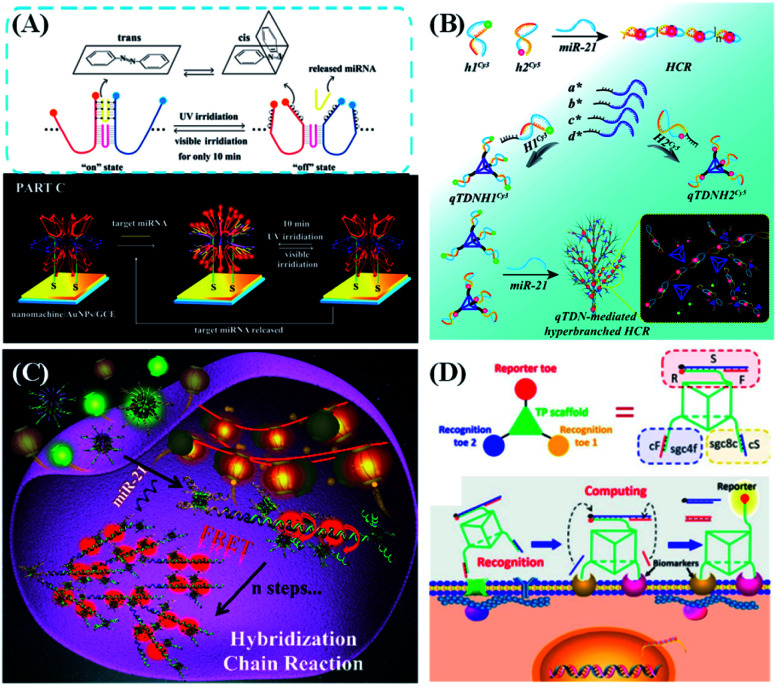
DNA nanostructure applied in biosensing. (A) 3D nanomachine-based electrochemical biosensors for rapid single-step quantitation of miRNA. Reproduced from ref. 109 with permission from the American Chemical Society, copyright 2018. (B) Tetravalent hairpin tetrahedron-mediated hyperbranched HCR for target miR-21 sensing. Reproduced from ref. 111 with permission from the Royal Society of Chemistry, copyright 2019. (C) Nanolantern-based DNA probe and signal amplifier for tumor-related biomarker detection. Reproduced from ref. 112 with permission from the American Chemical Society, copyright 2019. (D) A logic gate nanomachine for cancer cell-targeted imaging. Reproduced from ref. 113 with permission from the American Chemical Society, copyright 2018.

By anchoring DNA tetrahedron-based functional nucleic acid probes on the electrode surface, versatile electrochemical sensing platforms targeting small molecules, ions, exosomes and cells have been developed. Zheng *et al.*^107^ used tetrahedral DNA nanostructures and HCR amplification to detect DNA methylation. Three vertices of the DNA tetrahedron are modified with –SH groups and anchored on the gold disk electrode *via* the formation of Au–S bonds. The fourth vertex of the tetrahedron has a stem-loop capture probe, which can hybridize with the target ssDNA. If the target sequence is methylated, the HCR of biotin-labeled hairpin DNAs is initiated. *Via* the highly specific biotin–avidin interaction, multiple horseradish peroxidase (HRP) units are anchored on the electrode surface, catalyzing redox reactions to give a distinguishable electrochemical signal. Li *et al.*^108^ proposed a novel single-step electrocatalytic strategy called the blocker-assisted multibranched and bidirectional hybridization chain reaction (mbHCR). Through mutation inhibition and allosteric activation, the target binding affinity and HCR activity can be adjusted and programmed, thus realizing the one-step detection of multiple nucleic acids of diverse lengths. Besides DNA tetrahedrons, some other DNA nanostructures have also been modified on the chemical electrode. Chai *et al.*^109^ designed a 3D DNA nanostructure containing multiple azobenzene (azo)-functionalized DNA tweezers for rapid detection of miRNA in cancer cells in a single-step mode (Fig. 7A). Because the tweezers are evenly distributed on the DNA nanostructure, they are well organized and have a high local concentration, which improves the movement efficiency of the tweezers. Moreover, the photoisomerization of the azo groups leads to dehybridization/hybridization between the tweezers and the target (*e.g.*, miRNA) under irradiation with light of different wavelengths, thus realizing the process of dynamic regulation. Although the electrochemical methods are easy to operate and have low cost, it is still difficult for classic electrochemical DNA sensors to detect biomarkers at trace concentrations. In addition, target detection in complex samples will always suffer from inevitable background noise. Moreover, it is difficult to directly apply electrochemical biosensors in the detection of targets in living cells and tissues.

#### 4.1.2. Fluorescence sensing

Fluorescence is another signal output mode commonly used by DNA nanostructure-based biosensors. Due to the unique properties of DNA nanomaterials, different fluorophores and quenchers can be easily settled at designated sites with premeditated distances. In the process of target recognition, the distance will change, thus generating a switchable fluorescent signal. Fluorescent DNA biosensors show the advantages of extremely high sensitivity, good specificity, simplicity and low cost. Compared with electrochemical sensors, fluorescent sensors can be used for *in situ* real-time detection and imaging of biomolecules in cells. So far, they have been used in many fields such as the detection of nucleic acids, enzymes, proteins, pH and small molecule targets.^32,110^

Many reports have demonstrated that DNA nanostructure-based nucleic acid probes might show better sensing performance than traditional ones. For example, our group reported a 3D DNA tetrahedron-mediated hyperbranched HCR for the detection and imaging of cellular miR-21 (Fig. 7B).^111^ The traditional HCR is carried out by random diffusion of free DNA hairpins, which makes the kinetics and efficiency relatively low. In our design, each vertex of the tetrahedral DNA nanostructure assembles a DNA hairpin to construct a quadrivalent tetrahedral DNA nanoprobe (qTDN). This strategy can extend the HCR towards multiple reaction directions, increase the collision probability of probes, and achieve a high local probe concentration, thus greatly accelerating the reaction kinetics of the HCR. The reaction speed of the qTDN-mediated hyperbranched HCR is about 70 times fast than that of the traditional HCR. Due to the formation of hyperbranched HCR products, a stable FRET signal output can be obtained within 20 minutes, and high FRET efficiency up to 76% is obtained. Recently, our group prepared a multivalent DNA nanostructure, termed as DNA nanolantern (Fig. 7C).^112^ By combining with the HCR, the proposed DNA nanolantern-based FRET probe can be used as a signal amplifier to achieve the sensitive and specific imaging and detection of tumor-related RNA (including TK1 mRNA and miR-21) in living cells. DNA nanolanterns have significant advantages such as abundant and number adjustable probes, high FRET efficiency, good biocompatibility, and excellent biostability.

Besides target detection in solutions, DNA nanostructure-based nucleic acid probes were also demonstrated to work well on the cell surface.^114,115^ Tan's group reported an aptamer-based 3D DNA-logic gate triangular prism nanomachine as an intelligent system for cell surface logic gate calculation (Fig. 7D).^113^ The DNA triangular prism contains one reporter toe and two recognition toes. The two recognition toes can specifically recognize their membrane biomarkers on the human acute lymphoblastic leukemia (CCRF-CEM) cell surface *via* aptamer–target interactions, releasing two short ssDNAs to synergistically light up the fluorescence of the reporter's toe. Therefore, in the presence of CCRF-CEM cells, an “AND” logic operation is performed on the cell surface, giving an “ON” response to image the CCRF-CEM cells. Compared with freely dispersed dsDNA-based molecular circuits, this DNA triangular prism-based logic gate can easily integrate all logic units into an integrated DNA nanostructure, thus greatly improving the accuracy of cell identification.

In addition to electrochemical and fluorescent sensors, there are also some reports using the modulation changes in plasmonic nanostructures as the signal for sensing. In general, the modified gold nanorods were loaded onto DNA origami through complementary base pairing. After inputting signals such as RNA sequence and pH change, the plasmonic macromolecules were reconfigured.^116,117^ According to the change in distance or aggregation of the plasmon, different lights would be absorbed aligned with the direction of circular polarization, thereby generating a strong circular dichroism (CD) signal for detection.

### 4.2. Bioimaging

**Fig. 8:**
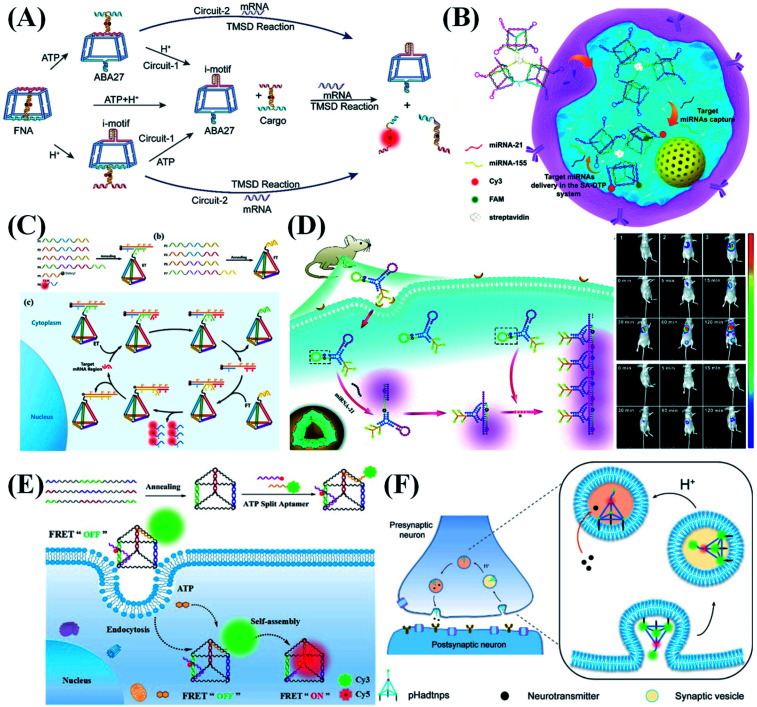
DNA nanostructure applied in bioimaging. (A) Environment recognizing DNA-computation circuits for mRNA imaging. Reproduced from ref. 119 with permission from Wiley-VCH, copyright 2020. (B) DNA prism frame structure for sensitive and multiplexed imaging of miRNAs in living cells. Reproduced from ref. 120 with permission from the American Chemical Society, copyright 2020. (C) Mechanism of the entropy-driven 3D DNA amplifier for intracellular mRNA imaging. Reproduced from ref. 121 with permission from the American Chemical Society, copyright 2018. (D) A tripartite DNA probe for RNA imaging in living mice *via* an HCR circuit. Reproduced from ref. 29 with permission from the Royal Society of Chemistry, copyright 2019. (E) DNA prism nanoprobes for ATP sensing in living cells. Reproduced from ref. 122 with permission from the American Chemical Society, copyright 2017. (F) Programmable pH-responsive DNA tetrahedron for imaging exocytosis and retrieval of synaptic vesicles. Reproduced from ref. 123 with permission from the American Chemical Society, copyright 2020.

DNA nanostructures are ideal bioimaging tools because of their good biocompatibility and self-assembly capabilities. So far, the bioimaging function of DNA nanomaterials has greatly promoted the development of early disease diagnosis and tissue imaging. Sensitivity, target recognition specificity, biocompatibility and biostability are the key to DNA nanostructure-based bioimaging applications. The basic principle of bioimaging is that the target (including specific nucleic acid sequences (DNA, mRNA, and miRNA), metal ions, proteins and small molecules) can be specifically recognized by probes, accompanied by the fluorescence signal changes (intensity change or FRET). Exploring the dynamics of endogenous RNA in living cells is a challenging experiment. Wong *et al.*^118^ designed a DNA tetrahedron transformer to display the abundance, distribution, and mobility of endogenous mRNA in living cells. By combining single-particle tracking, single-molecule fluorescence *in situ* hybridization (FISH) and colocalization research, they studied the dynamic changes of delta-like ligand 4 (DLL4) mRNA that regulates the formation of tip cells during angiogenesis. Combining the advantages of functional nucleic acids and logic circuits, Li *et al.*^119^ built two cascade logic circuits, OR–AND and AND–AND, for cellular DNA calculation and subcellular mRNA imaging (Fig. 8A). The I-motif structure and ATP aptamers embedded in a truncated square pyramid cage act as logical control units, in response to intracellular H^+^ and ATP, triggering the release of the sensing element to achieve the target mRNA imaging.

To improve the detection sensitivity of nucleic acid probes, appropriate signal amplification techniques are usually elegantly introduced in the corresponding sensing systems. To overcome the difficulty of transporting biological enzymes into living cells, some enzyme-free signal amplification techniques, such as the HCR, CHA and entropy-driven signal amplifier have been designed for intracellular signal amplification, and they are also demonstrated to work well for DNA nanostructure-mediated intracellular imaging analysis. Inspired by the tentacles of an octopus, Zhang *et al.*^120^ prepared a multivalent DNA triangular prism (DTP), which is assembled by linking three biotinylated DTPs by a streptavidin for sensitive, rapid and multiplexed imaging of miRNAs in living cells (Fig. 8B). In the presence of miR-155 and miR-21 targets, two CHA reactions are successively initiated, achieving the precise imaging analysis of aberrant expression and dynamic change of target miRNAs in different cells. Due to the increase in local probe concentrations, the two CHA reactions show obviously accelerated kinetics. As a newly emerged enzyme-free signal amplification technique, entropy-driven signal amplification also shows great promise for intracellular applications. Tan *et al.*^121^ reported an entropy-driven 3D DNA tetrahedral amplifier that can identify and image specific mRNA in living cells (Fig. 8C). Due to the unique entropy driving force, an autonomous DNA circuit can be specifically activated in living cells, thereby providing huge signal amplification for ultra-sensitive detection and imaging of mRNA. In addition to entropy-driven amplifiers, the HCR is also widely used in the field of bioimaging. Jiang *et al.*^29^ reported a Y-scaffold-based tripartite DNA probe for fluorescent RNA imaging in living mice through a HCR circuit (Fig. 8D). This tripartite probe can be selectively and efficiently internalized into cells overexpressing folic acid receptors through endocytosis, achieving a high-contrast image for miR-21 in live mice.

In addition to the detection of nucleic acids, DNA nanostructure-based fluorescent probes have also been used for detection of other biomolecules. For example, accurate *in situ* monitoring of telomerase activity is of great significance in the early diagnosis of cancer. The major challenge faced by telomerase detection in living cells is the complex intracellular biological environment, which makes it difficult for traditional fluorescent probes to achieve effective cell penetration, stable signal output and accurate intracellular imaging. Meng *et al.*^124^ designed a tetrahedral DNA nanoprobe integrated with a structure-switchable molecular beacon to detect and image telomerase in living cells. Telomerase-catalyzed extension of the nanoprobe primer strands triggers a strand displacement reaction, releasing free molecular beacons and thus giving increased FRET signals. The proposed tetrahedral nanoprobe can accurately detect telomerase activities in different cells and distinguish cancer cells from normal cells. Zhang *et al.*^122^ prepared a DNA prism containing a split aptamer for sensing ATP in living cells (Fig. 8E). In the presence of ATP, the two parts of the split aptamer simultaneously bind to ATP, bringing Cy3 and Cy5 fluorophores into close proximity and thus resulting in high FRET efficiency. Because the DNA nanostructure can strongly protect the split aptamer from nuclease degradation, reliable imaging analysis of ATP expression in living cells can be achieved *via* a “FRET-OFF” to “FRET-ON” mode.

The detection of intracellular metal ions is of great significance for understanding intracellular metal homeostasis and related diseases. Xiang *et al.*^125^ prepared a two-color coded DNAzyme tetrahedral nanoprobe for sensitive detection of UO_2_^2+^ and Pb^2+^ in living cells. UO_2_^2+^ and Pb^2+^ can cleave their respective substrate sequences in the DNAzyme-encoded nanostructure, lighting up the corresponding fluorescence signal. Such a sensing platform can be used for the simultaneous ultra-sensitive detection of UO_2_^2+^ and Pb^2+^ with detection limits of 0.6 nM and 3.9 nM, respectively. In addition, by combining DNA nanotechnology with DNA computation, DNA nanostructure-based molecular logic gates were constructed for multi-parameter sensing, molecular computing, and intelligent diagnosis.^66^

Besides target detection in living cells, DNA nanostructure-based nucleic acid probes can also be used for the study of physiological action. Tan *et al.*^123^ reported a programmable pH-responsive ratiometric DNA tetrahedral nanoprobe for imaging exocytosis and extraction of synaptic vesicles (Fig. 8F). Three vertices of the DNA tetrahedron are labeled with cholesterol molecules, which can effectively and stably anchor the tetrahedron to the neuronal plasma membrane. According to the change of lumen pH during the synaptic vesicle cycle, the tetrahedral probe can trace its exocytosis and recovery status in real-time by monitoring the changes in the fluorescence ratio of two fluorophores.

### 4.3. Cell assembly and capture

**Fig. 9:**
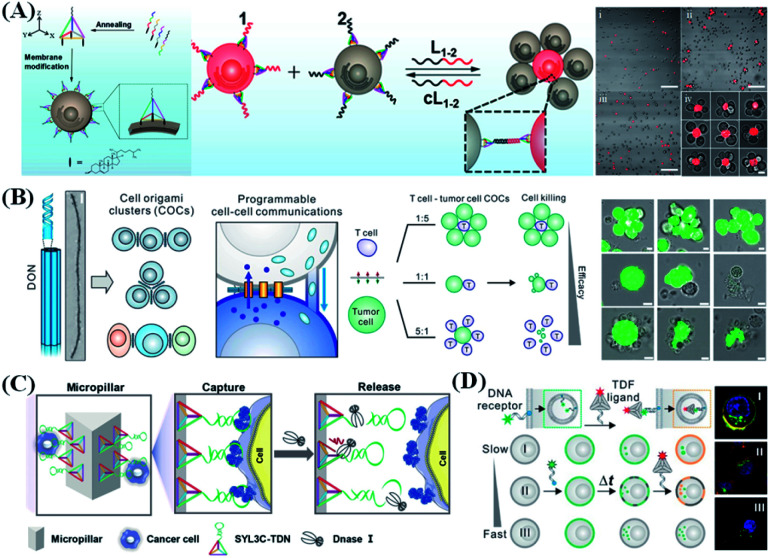
DNA nanostructure applied in cell assembly and capture. (A) Cell membrane-anchored DNA tetrahedron for cell assembly. Reproduced from ref. 126 with permission from the American Chemical Society, copyright 2019. (B) Programming cell–cell communications assembled with engineered cell origami clusters. Reproduced from ref. 127 with permission from the American Chemical Society, copyright 2020. (C) DNA nanolithography in a microfluidic chip for efficient capture and release of CTCs. Reproduced from ref. 128 with permission from Wiley-VCH, copyright 2020. (D) Discrimination of different cell lines by monitoring DNA tetrahedron-encoded ligand–receptor interactions on the cell membrane. Reproduced from ref. 129 with permission from the American Chemical Society, copyright 2020.

Precise control of cell–cell interaction allows straightforward study and manipulation of various cellular processes. Cells communicate with others mainly through the components located on the membrane or released outside the cells as mediators. Therefore, designing functional modules with high controllability and stability after anchoring on the cell surface will provide a good opportunity to study and manipulate the intercellular reactions. DNA nanostructures provide excellent tools for precisely controlling the distance and orientation of cell interactions. Tan *et al.*^126^ reported an amphiphilic tetrahedral DNA probe for cell surface engineering (Fig. 9A). One vertex of the tetrahedron has an extended DNA recognition probe, and the remaining vertices were modified with cholesterol. Based on hydrophobic insertion, cholesterol molecules can be quickly and effectively anchored on the phospholipid layers of the cell membrane. Moreover, their membrane anchoring affinity can be fine-tuned by adjusting the number of cholesterol molecules. Compared to linear DNA probes, these tetrahedral probes show higher membrane anchoring stability (by nearly 100 times) and higher target accessibility (by about 2.5 times). The use of DNA nanostructures can help scientists achieve specific, effective and adjustable control of cell adhesion. However, the assembly of cells in a spatial orientation remains to be explored. Fan *et al.*^127^ reported a DNA origami nanostructure to assemble homogeneous and heterotypic cells in 3D space (Fig. 9B). Compared with single-stranded molecular probes, DNA origami molecular probes are more rigid and can provide a specified type, number, and direction of anchor points, so they are not easily internalized by cells and can settle stably on the cell membrane for a long period. By utilizing the nanoclusters assembled by homogeneous and heterogeneous cells, three different types of cell–cell communication can be investigated, including gap junctions, tunnel nanotubes, and immune/tumor cell interactions.

The controlled capture and release of specific cells play important roles in tissue engineering, disease therapy and biomedicine. In order to achieve efficient capture and release of cells, some classic strategies such as flow cytometric cell sorting and immunomagnetic enrichment have been developed. Those strategies usually have high cost and are time consuming. DNA nanomaterials are also widely used for cell capture and release due to their excellent anchoring and integration capabilities. Yang *et al.*^130^ developed a new strategy for capturing stem cells by physically cross-linking DNA networks that are prepared by double RCA reactions. The DNA network creates a flexible, programmable and biocompatible 3D system for cell enveloping and separation *via* specific recognition of aptamer units in the network to stem cells. In addition, the physically cross-linked DNA network does not cause significant damage to the cells, thus maintaining the cell activity for further biological analysis. The Li and Zuo groups used DNA hydrogel, which was prepared by the HCR, to encapsulate and release living tumor cells.^131^ They introduced an aptamer-trigger-clamped hybridization chain reaction (atcHCR) method to form DNA hydrogel by using the DNAs specifically bound on the surface of circulating tumor cells (CTCs) as initiators. Subsequently, the captured CTCs could be released by ATP because the ATP molecule would destroy the DNA hydrogel by causing the conformational change of the aptamer units in the DNA hydrogel. By anchoring different numbers of aptamers on the vertices of the DNA tetrahedron or tetrahedron dimer, Zhu *et al.*^103^ generated a family of tetrahedral DNA frameworks (TDFs) containing topologically controlled ligands to regulate the binding strength between ligands and receptors. In this way, cell sorting from a cell mixture was achieved with minimum cell damage. Enlightened by the synergistic effect adapted by the immune system, Li and Zhang's group^132^ developed a bioinspired DNA nanointerface (named as DNA nanosynapse) by coupling three different aptamers to the three vertices of the DNA tetrahedron. *Via* the simultaneous binding of the three aptamers to their respective target proteins on the CTC surface, the DNA nanosynapse can persistently adhere to the membrane of CTCs, thus showing strong resistance toward endocytosis and making it a promising tool for CTC capture, detection and downstream disease status analysis.

Microfluidic chips modified with DNA nanostructures have shown great potential in capturing biomolecules and cells. By anchoring a planar triangle of the DNA tetrahedron on the chip surface and adding an aptamer to the top vertex of the DNA tetrahedron, Yang *et al.*^128^ reported a novel aptamer-modified microfluidic chip (Fig. 9C) to improve the capture efficiency. The rigid tetrahedral DNA scaffold provides a highly ordered vertical orientation and controllable spacing for the aptamers, which is helpful for the aptamers to effectively recognize the target cells and avoids the undesirable orientation or crowding effect faced by the traditional microfluidic interface manufacturing process. In addition, due to the vertical orientation of the aptamer, it is more prone to hydrolysis by deoxyribonuclease I (DNase I), resulting in a cell release rate of up to 83% and a cell survival rate of up to 91%. In recent years, Zhu *et al.*^129^ also developed a DNA-encoded receptor-ligand system to monitor cell membrane-associated redistribution of molecules and thus to classify different cell types (Fig. 9D). In this system, a cholesterol-modified ssDNA serves as the receptor on the membrane, and a tetrahedral DNA backbone with complementary ssDNA hangings serves as the ligand. Because different cell membrane redistribution dynamics lead to distinct ligand–receptor interaction patterns, different cell lines can be discriminated in this way.

### 4.4. Theranostics

In recent decades, various cancer treatments have been extensively studied to tackle the rising incidence and mortality of cancer. To achieve targeted therapy and thus to improve therapeutic efficacy, drug delivery systems have been widely developed. Inorganic nanomaterials, liposomes, and polymers are all encouraging drug carrier candidates. However, immunotoxicity, exogenousness, and design complexity are the major issues that hinder their applications. As a kind of biological material, DNA has its unique advantages in biomedical applications because of its good biocompatibility, easy modification and programmability. DNA nanostructures with a diameter range from nanometer to micrometer have been proven to have high cell uptake efficiency,^3^ which provides new ideas for DNA nanostructure-based cancer treatment methods. Compared with simple nucleic acids, DNA nanostructures have high flexibility in different sizes, allowing adjustable cargo capacity. At the same time, programmable nanostructures can be modified with functional nucleic acids for precise positioning and targeted therapy. So far, many researchers have used DNA nanostructures to transport various types of drug molecule or cargo into target cells, including chemotherapeutic drugs, photosensitizers, CpG oligonucleotide, siRNA, Cas9 and antibodies.^7^ In this section, we will summarize the latest advances in DNA nanostructures for various cancer therapies, including chemotherapy, immunotherapy, photodynamic therapy and gene therapy.

#### 4.4.1. Chemotherapy

**Fig. 10:**
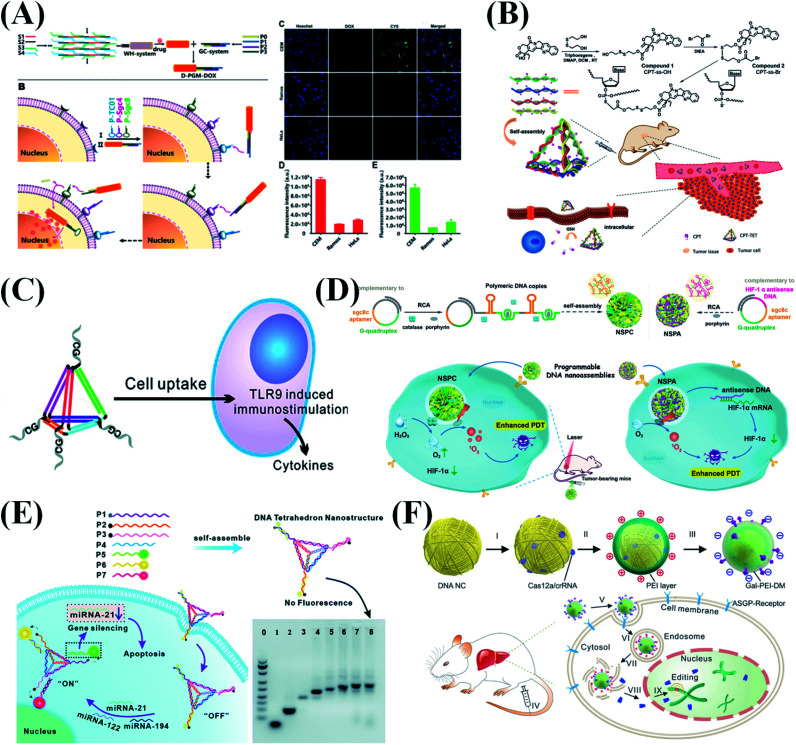
DNA nanostructure applied in cancer therapy. (A) Precision-guided missile-like DNA nanostructure for aptamer-based targeted drug delivery into cancer cells. Reproduced from ref. 135 with permission from the American Chemical Society, copyright 2020. (B) The CPT-grafted DNA tetrahedron as a stoichiometric nanomedicine for tumor therapy. Reproduced from ref. 136 with permission from Wiley-VCH, copyright 2019. (C) Self-assembled immunostimulatory CpG oligonucleotide DNA nanostructures for immunotherapy. Reproduced from ref. 137 with permission from the American Chemical Society, copyright 2011. (D) Programming DNA nanoflower for enhanced photodynamic therapy. Reproduced from ref. 92 with permission from Wiley-VCH, copyright 2020. (E) DNA tetrahedron for accurate cancer identification and miRNA silencing induced therapy. Reproduced from ref. 138 with permission from the Royal Society of Chemistry, copyright 2019. (F) RCA-based DNA nanoflower for the delivery of CRISPR/Cas12a RNA to regulate serum cholesterol levels. Reproduced from ref. 94 with permission from the American Association for the Advancement of Science, copyright 2020.

Chemotherapy is a widely used drug-related therapy in clinical tumor treatment. Chemotherapy kills cancer cells by delivering specific drugs such as doxorubicin (DOX), fluorouracil (FU), and camptothecin (CPT) to tumor tissues. However, the disadvantages of using chemotherapeutic drugs are unsatisfactory therapeutic efficacy and adverse side effects. It is essential to deliver anti-cancer drugs to target cells with high precision and high efficiency. DNA nanostructures functionalized by specific groups to construct targeted drug delivery systems might perfectly solve this problem. Many chemotherapy drugs can be coupled with DNA through covalent and non-covalent bonds. For example, DOX can effectively intercalate into GC-rich dsDNA.^133,134^ In addition, DNA nanomaterials can also integrate functional nucleic acid aptamers (for example mucin 1 protein (MUC1) aptamer, nucleolin aptamer AS1411 and protein tyrosine kinase 7 aptamer sgc8) to enhance targeting specificity, and significantly reduce adverse side effects. Wu *et al.*^135^ reported a DNA nanoscale precision-guided missile to efficiently load and accurately deliver chemotherapy drugs to target cells (Fig. 10A). In their design, rod-shaped nanostructures modified with three different aptamers serve as drug carriers. As an integrated logic gate, the three aptamers can perform cell subtype-specific recognition through sequential disassembly mediated by cell-anchored aptamers.

Most chemotherapeutic drugs are hydrophobic molecules that are poorly dissolved and dispersed in the aqueous environment of a living system. Therefore, it is necessary to design suitable carriers to deliver the drugs to the desired positions. Based on the good hydrophilicity and programmability of DNA, Zhang *et al.*^136^ constructed a CPT-grafted DNA tetrahedron as a precise nanodrug to inhibit tumor growth (Fig. 10B). Considering the high tumor recurrence rate and possible residual tumor cells after surgery, Zhang *et al.*^139^ reported a DNA hydrogel grafted with CPT to prevent tumor recurrence. A large number of CPT molecules were successfully grafted onto the phosphorothioate DNA backbone of two types of Y-shaped building block, which were then assembled into drug-containing hydrogels *via* “sticky-end” associations of building blocks.

Generally, DNA nanomedicine is administered by invasive needle injection. In such a systemic drug delivery process, in addition to clearance by the immune system and kidneys, DNA nanostructures also face rapid disintegration and digestion by nucleases. All of this might result in reduced drug availability at the target site. Xu *et al.*^140^ reported a transdermal drug delivery method that can avoid the above-mentioned shortcomings. The transdermal drug administration method has the advantages of minimal invasiveness and the lack of need to participate in the internal circulation or liver metabolism. It can achieve the expected therapeutic effect with minimal side effects. Using the mouse melanoma model, it was proved that the DOX released by the DNA tetrahedron carrier was 2-fold that released by other inorganic carriers.

#### 4.4.2. Immunotherapy

Immunotherapy is also an effective strategy for cancer therapy, mainly through stimulating and activating the host immune system to treat tumor cells.^141^ A cytosine–phosphate–guanine (CpG) dinucleotide is a therapeutic nucleic acid with strong immunostimulatory activity. The CpG motif can be recognized by toll-like receptor 9 (TLR9) after being taken up by cells, producing high levels of secretion of various proinflammatory cytokines, including tumor necrosis factor (TNF)-R, interleukin (IL)-6 and IL-12. Such stimulation-induced immunology method has been widely used as an effective strategy for treating tumor cells.^142^ By attaching an unmethylated CpG motif to 3D DNA tetrahedrons, Fan *et al.*^137^ developed functional multivalent DNA nanostructures for immune activation therapy (Fig. 10C). These nanostructures can be efficiently and non-invasively taken up by RAW264.7 cells. They remain essentially intact in cells for 8 hours and efficiently induce intracellular secretion of cytokines after being taken up. Chang *et al.*^143^ reported an RNA origami nanostructure for efficient and safe anti-cancer immunotherapy. This RNA origami nanostructure can function as a strong toll-like receptor 3 (TLR3) agonist to stimulate a potent innate response. The structure is stable, economically feasible, effective, safe, and is an ideal drug for *in vivo* applications. During the delivery process, DNA nanostructures may denature under physiological salt concentration and be degraded by nucleases. More seriously, undesirable immune responses (*e.g.*, potential DNA-induced inflammatory effects) might be caused by DNA nanostructure-based delivery systems.^144^ Kostiainen *et al.*^145^ reported that the special protein coating of DNA nanostructures can enhance stability and immunocompatibility. The BSA-based coating of DNA nanostructures was shown to attenuate the immune activation of mouse primary splenocytes. Scientists also reported that the use of viruses and oligolysine as a shield for DNA nanostructures can improve their stability *in vivo* and reduce the immune response.^146,147^ Recently, more and more information about the cGAS-STING pathway has been revealed.^148^ Considering that this pathway explains the mechanism of how exogenous DNA can be recognized and how immunological stress can be initiated, the DNA-based nanostructure probably provides a potential tool for immunotherapy through the cGAS-STING pathway.

#### 4.4.3. Photodynamic therapy

Photodynamic therapy (PDT) is a highly efficient and non-invasive therapy that utilizes the vulnerability of cells to reactive oxygen species (ROS) to treat cancer and some other diseases.^149–151^ Because PDT only causes lesions through controllable irradiation, it avoids the side effects of systemic treatment. As an indispensable element, a photosensitizer plays a vital role in PDT.^152^ However, due to the low water solubility and small size of photosensitizers, they often find it difficult to enter cancer cells. A good carrier can improve photosensitizer delivery efficiency and overcome membrane barriers. DNA nanocarriers have been widely used in photodynamic therapy due to the efficient loading of photosensitizers. To solve the problem that the inherent hypoxic tumor microenvironment limits the efficacy of PDT, Liu *et al.*^92^ reported a multi-functional DNA nanorod, which is constructed by the RCA reaction, for effective co-loading and delivery of photosensitizers, catalase and hypoxia-inducible factor 1α (HIF-1α) antisense DNA (Fig. 10D). Catalase can catalyze the *in situ* production of O_2_ to relieve the hypoxic tumor microenvironment, and antisense DNA can downregulate HIF-1α to enhance PDT efficacy. Liang *et al.*^153^ used a self-assembled 3,6-bis 2-[(1-methylpyridinium)ethynyl]-9-pentylcarbazole diiodide (BMEPC) photosensitizer-loaded DNA origami nanosystem as a new type of dual-function therapeutic agent for highly efficient PDT. The DNA origami nanostructure effectively protects BMEPC from photobleaching after being irradiated, thus endowing the photosensitizer molecule with higher PDT efficacy than that of free BMEPC.

#### 4.4.4. Gene silencing

Gene therapy usually involves introducing healthy genes or gene regulators into abnormal cells to prevent or treat diseases. The current gene therapy protocols can be mainly divided into antisense DNA, RNA interference (RNAi), and CRISPR/Cas gene-editing methods. The antisense DNA strand is complementary to the target gene, and it can inhibit gene expression or translation. The RNAi method uses small interfering RNA (siRNA)^47,154^ or short hairpin RNA (shRNA)^155^ to recognize and cleave target mRNA. However, monomeric siRNA, shRNA, and antisense DNA strands are difficult to internalize into cells. So scientists have developed many tools to deliver them to targets. DNA nanostructures have high loading capacity and biocompatibility and are considered to be ideal nucleic acid carriers. By using a DNA tetrahedron to assemble three recognition sequences for target miRNAs and one recognition sequence (antagomir-21) for miR-21, Liu *et al.*^138^ developed a smart nanosystem for simultaneous monitoring of three intracellular miRNAs and meanwhile inducing cancer cell apoptosis by silencing endogenous miR-21 inside cells (Fig. 10E).

Chen *et al.*^155^ performed *in situ* synthesis of shRNA on the surface of amphiphilic DNA-polylactide micelles and used the obtained nanoparticles to carry DOX. The as-prepared nanomedicine enables efficient co-delivery of nucleic acid therapeutics and chemotherapeutics to xenograft tumors, leading to obvious multidrug resistance protein 1 (MDR1) gene expression inhibition, enhanced intracellular accumulation of DOX, and potentiated apoptosis, and thus improved tumor treatment efficacy.

CRISPR–Cas9 represents a promising genome editing tool, but it requires a safe and efficient delivery platform to be effectively transported to the lesion site. Gu *et al.*^156^ synthesized a yarn-shaped DNA nanoclew by RCA and used it to deliver CRISPR–Cas9 for targeted genome editing. The partial complementarity between the DNA nanoclew and the sgRNA guide sequence greatly improves the degree of gene editing because the nanoclew's binding and the release of the Cas9/sgRNA complex have been balanced. They further reported that the DNA nanoclew could be used to deliver CRISPR/Cas12a RNA to regulate serum cholesterol levels (Fig. 10F).^94^ Zhang *et al.*^105^ used extracellular vesicles engineered by valence-regulated DNA nanostructures to carry the CRISPR/Cas9 system for gene therapy. A valence-regulated DNA tetrahedron is designed to combine with DNA aptamers, and specific cell targeting can be achieved by adjusting different aptamer/cholesterol ratios (1 : 3–3 : 1) in the tetrahedron.

## 5. Conclusions and perspectives

Self-assembled DNA nanostructure based nucleic acid probes have shown great potential in biosensors, drug delivery, cell biology, and material nanofabrication. In this review, we have summarized several typical DNA nanostructures from different dimensions and discussed their recent research progress and biological applications. Nucleic acid probes have various functions such as molecular recognition, catalytic activity and therapeutic effects. However, naked single-stranded nucleic acid probes show inefficient cell internalization due to their small size and negatively charged backbone. Once they enter the cells, they are also easily degraded by nucleases before reaching the target organelle. In recent years, nanocarriers have been widely used to facilitate DNA probes in cellular applications, which have brought new opportunities for their application in the field of biomedicine and bioanalysis. Studies have demonstrated that self-assembled DNA nanostructure based nucleic acid probes have higher cell uptake efficiency and lower nuclease degradation than nucleic acid probes alone. Various programmed DNA nanostructures have been used for biomarker detection, cell imaging, and *in vivo* diagnosis. Through Watson–Crick base pairing or click chemistry, nucleic acid probes can be loaded onto self-assembled DNA nanostructures, and respond to external stimuli such as molecular targets, pH, temperature and so on. Various targets ranging from nucleic acids, metal ions, and proteins to cancer cells and living organisms can be specifically recognized by particular DNA probes. DNA nanostructure based delivery systems can carry chemotherapy drugs, photosensitizer molecules, CpG and gene therapy nucleic acids for targeted therapy. These platforms have been proven to have improved cell permeability, high biocompatibility, good robustness, smart responsiveness, and targeted cellular uptake.

Despite the impressive progress and high expectations, the development of DNA-based structures/technologies is still hampered by several recognized challenges. (i) The chemical synthesis of long DNA sequences (>100 nt) is still difficult and expensive. Large-scale nanostructures such as DNA origami structures or DNA bricks usually require hundreds of ssDNA strands, which further increases the total costs. In addition, the large-scale production and purity of self-assembled DNA nanostructures still need to be improved, which may affect the effectiveness and accuracy of the biosensors. (ii) Although DNA nanostructures show better resistance to enzymatic hydrolysis than ssDNAs, they are not sufficient for long-term incubation. DNA nanostructures usually require extremely high concentrations of Mg^2+^ to maintain structural integrity, which is too high to cellular microenvironment. (iii) Although some multivalent nanostructures have been proposed to improve the ability for target recognition, there is still a need to develop more stable nanostructures with more anchor points. (iv) The immune response triggered by the DNA nanostructure delivery system may also limit its application in biomedicine.^144^ Whether artificially designed DNA nanostructures can cause genetic diseases is also lacking in studies. (v) DNA nanostructures can deliver other molecules into the cytoplasm of living cells, but they still need to be improved for the targeting of particular organelles. This largely prevents the deciphering of intracellular molecular events. (vi) In terms of drug delivery, high tumor targeting and low cytotoxicity are the advantages of DNA vectors, most of which require functional modification. However, this may be a double-edged sword, meaning that functionalization improves the performance of DNA vectors, and large-scale modification may hinder the effective loading and release of drugs. In addition, the pharmacokinetics, circulation, metabolism and excretion of the nanostructures after loading in the biosystem have not been fully studied.

Looking ahead, the construction and application of DNA nanostructure-based nucleic acid probes will keep evolving. For example, some strategies have been explored, including asymmetric PCR, RCA, and fermentation to solve the problem of low productivity. DNA components formed by gene expression can produce large-scale, high-purity nanostructures, which are convenient for application *in vivo*. Using bacteriophages to generate single-stranded precursor DNA can also reduce the high total cost of DNA origami.^157^ Although the rigid DNA nanostructures showed enhanced resistance to nuclease degradation compared with duplexes or plasmid DNA, this may not be sufficient for practical applications. Recently, several strategies have been developed to increase the nuclease resistance of DNA nanostructures while retaining their functions.^158–160^ Through enzymatic ligation, click chemistry and photo-crosslinking methods, the ends of the DNA strands are joined or cross-linked for eliminating internal gaps. Reducing the number of free ends could effectively enhance the nuclease resistance of the DNA nanostructures. Some of the macromolecules such as proteins, lipids and polyelectrolytes can interact electrostatically with the negatively charged DNA phosphate backbone, forming protective shells on the surface of DNA nanostructures. Many kinds of shell have been proposed to reduce the immune response, promote internalization, and increase the nuclease resistance of DNA nanostructures. Therefore, these modifications have been studied on the stability of various DNA nanostructures in biological fluids such as serum, urine and cell lysates. To improve the stability, sensitivity and accuracy of biosensors, DNA nanomaterials can be combined with signal amplification strategies or other nanomaterials (such as gold nanoparticles, graphene oxide and quantum dots) to achieve ultra-sensitive detection. At the same time, we should embrace actual needs, deeply integrate DNA nanostructures into different fields, and explore their potential applications in smart drug delivery vehicles, nanorobots, nanomechanical equipment, energy storage, and materials science. With the significant increase in the applications of self-assembled DNA nanostructures, as well as that in other fields, we are optimistic that these potential tools will have broad application prospects soon.

## Conflicts of interest

There are no conflicts to declare.
